# Copper Homeostasis in the Model Organism *C. elegans*

**DOI:** 10.3390/cells13090727

**Published:** 2024-04-23

**Authors:** Verena Alexia Ohse, Lars-Oliver Klotz, Josephine Priebs

**Affiliations:** Nutrigenomics Section, Institute of Nutritional Sciences, Friedrich-Schiller-Universität Jena, 07743 Jena, Germany; verena.ridolfi@uni-jena.de

**Keywords:** *C. elegans*, copper transport, copper deficiency, copper toxicity, reactive oxygen species

## Abstract

Cellular and organismic copper (Cu) homeostasis is regulated by Cu transporters and Cu chaperones to ensure the controlled uptake, distribution and export of Cu ions. Many of these processes have been extensively investigated in mammalian cell culture, as well as in humans and in mammalian model organisms. Most of the human genes encoding proteins involved in Cu homeostasis have orthologs in the model organism, *Caenorhabditis elegans* (*C. elegans*). Starting with a compilation of human Cu proteins and their orthologs, this review presents an overview of Cu homeostasis in *C. elegans*, comparing it to the human system, thereby establishing the basis for an assessment of the suitability of *C. elegans* as a model to answer mechanistic questions relating to human Cu homeostasis.

## 1. Introduction

Copper is an essential trace element that is involved in a multitude of biological pathways in humans, mostly as part of copper proteins that are crucial to processes as diverse as mitochondrial respiration (e.g., cytochrome *c* oxidase), antioxidant defense (e.g., Cu,Zn-superoxide dismutase), iron homeostasis (e.g., ceruloplasmin, hephaestin), xenobiotic metabolism (e.g., copper-dependent amine oxidases), connective tissue biogenesis (e.g., lysyl oxidase) and the generation of tyrosine metabolites (e.g., tyrosinase), to name a few (for review, see [1]; Table 1). All of these examples of copper-dependent enzymes are oxidoreductases, highlighting the role of copper in biological redox reactions, with cuprous (Cu^+^) and cupric (Cu^2+^) ions being the predominant forms of Cu in biological systems [1]. (When mentioning “copper” or “Cu” in this article, such as in “Cu homeostasis”, this does not refer to the metal, but rather cuprous and cupric ions).

It is this very nature of copper ions that can also contribute to Cu toxicity, in that its redox activity may result in the generation of potentially harmful reactive oxygen species (ROS), e.g., through Fenton-type reactions or redox cycling [2,3]. A second mode of interfering with biological processes, is, of course, through the interaction with biomolecules, such as proteins: for example, according to the ‘‘hard and soft acids and bases’’ concept [4], Cu^+^, as a ‘‘soft’’ cation, will avidly interact with ‘‘soft’’ thiolates of cysteinyl residues in proteins.

*Caenorhabditis elegans* (*C. elegans*) lives and proliferates in soils in temperate climates, as well as compost and other environments characterized by decomposing fruits and plant material [5]; it feeds on bacteria and has been in use as a model organism in molecular and cell biology research laboratories since its introduction into that field by Sydney Brenner [6]. In its natural habitat, *C. elegans* is exposed to strongly fluctuating levels of environmental compounds, including metal (such as copper) ions. Moreover, more than 50% of human protein-coding genes (numbers range from 60–80%, in older predictions [7], to approx. 53%, according to “OrthoList2” [8]), including many genes associated with human diseases, have an ortholog in *C. elegans*. It is no wonder, then, that similarities between humans and *C. elegans* also exist at the level of Cu homeostasis, including copper proteins, as well as Cu uptake, Cu distribution and Cu excretion mechanisms.

In order to provide a background with respect to whether, and to what extent, *C. elegans* can be useful as an in vivo model for research into mammalian Cu homeostasis, this review will summarize Cu homeostasis in *C. elegans*, compare it with human Cu homeostasis and discuss the adaptation to copper toxicity and deficiency in nematodes.

## 2. The Human Copper Proteome and Orthologs in *C. elegans*

If a portion of human genes as large as that stated above have orthologs in *C. elegans*, this would also include genes encoding copper-related proteins. In humans, several dozen of Cu-related proteins have been identified so far, including Cu-dependent enzymes, Cu transporters and other Cu-related proteins. Most of these have orthologs in *C. elegans*, but the role of these orthologs in nematode Cu homeostasis is not always clearly defined. In order to allow for a discussion of Cu homeostasis in *C. elegans* and a comparison with the human system, we collated information on the human Cu proteome and searched for orthologs in *C. elegans*. Building on the work by Blockhuys et al. [9], who identified 54 human Cu-binding proteins and allocated each to one of three categories (enzymes, transporters and proteins with unknown or other functions), we used similar categories and listed known human Cu proteins, together with information on their (potential) orthologs in *C. elegans*. These data are listed in Table 1, Table 2 and Table 3, divided by category: (i) Cu enzymes, (ii) Cu chaperones and transporters, (iii) Cu buffers and other Cu-binding proteins.

Our tables have nine entries more than the previously published list [9], five of which are more recently identified Cu proteins: Mucin (MUC) 2 (and, by extension, MUC5AC and MUC5B) [10], selenium-binding protein 1 (SELENBP1; see Section 3.3. below) [11], and a mitochondrial Cu transporter, SLC25A3 (see Section 3.2.2. below) [12]. Further additions to the list are due to the inclusion of metallothioneins 1 and 2, as they may also bind Cu under conditions of Cu excess [13] (hence, their inclusion in the table listing Cu buffers); and the amyloid precursor-like protein (APLP) 2, which, next to the amyloid precursor protein (APP), has a functional Cu-binding domain [14]. Adenosylhomocysteinase (AHCY) binds copper strongly, but it does not appear to be required for enzymatic activity (for review, see [15]); the protein is, therefore, listed in Table 3 (“other”). Another protein, previously listed as a Cu enzyme by Blockhuys et al. [9], was—based on recent publications by the same group, demonstrating a protective effect of MEMO1 based on Cu sequestration [16,17]—listed under buffer/other (Table 3); the interaction with, and the transfer of Cu from MEMO1 to ATOX1 that was shown to occur in vitro [16], may result in it being considered a chaperone rather than buffer.

It is evident from Table 1, Table 2 and Table 3 that several human Cu proteins do not have orthologs in *C. elegans*. No ortholog was identified or predicted for 19 of the 63 human proteins. This does not always exclude the presence of functional orthologs that are not Cu proteins. For example, a manganese-containing superoxide dismutase could be considered a functional ortholog of Cu,Zn-superoxide dismutase, as the catalyzed reaction is the same. Moreover, it does not necessarily mean that the number of Cu proteins is lower in nematodes compared to humans, as the tables reflect a unidirectional search for orthologs; the purpose of this article is to assess to what extent the human Cu proteome is mirrored in the model organism *C. elegans*. Nevertheless, we also tested whether human orthologs of *C. elegans* Cu proteins not listed in Table 1, Table 2 and Table 3 exist.

**Table 1:** Human Cu proteins and their *C. elegans* orthologs (I): Cu-dependent enzymes.

Enzyme Activity	HGNC Symbol ^a,b^	UniProt-ID ^c^	*C. elegans* Gene Ortholog ^b,d,f^	Reference ^e^
Amine oxidase	AOC1	P19801	no ortholog	[a], [b]
	AOC2	O75106		
	AOC3	Q16853		
Cytochrome *c* oxidase	MT-CO1	P00395	*ctc-1/cox-1*	[a], [b]
	MT-CO2	P00403	*ctc-2/cox-2*	[a], [b]
Dopamine β-hydroxylase	DBH	P09172	*tbh-1*	[a], [b]
	MOXD1	Q6UVY6		[a], [b]
	MOXD2P	A6NHM9		[b]
Ecto-NOX disulfide-thiol exchanger	ENOX1	Q8TC92	no ortholog	[a], [b]
	ENOX2	Q16206		
Ferroxidase	CP	P00450	F21D5.3	[a], [b]
	HEPH	Q9BQS7		
	HEPHL1	Q6MZM0		
Lysyl oxidase	LOX	P28300	no ortholog	[a], [b]
	LOXL1	Q08397	no ortholog	[a], [b]
	LOXL2	Q9Y4K0	C06B8.7	[b]
	LOXL3	P58215	C06B8.7	[b]
	LOXL4	Q96JB6	no ortholog	[a], [b]
MAPK/ERK kinase	MAP2K1	Q02750	*mek-2* *	[a], [b], [18]
Methanethiol oxidase	SELENBP1	Q13228	*semo-1* *	[19]
Peptidyl-glycine α-amidating monooxygenase	PAM	P19021	*pamn-1*, *pgal-1*, *pghm-1*	[b]
Protein deglycase	PARK7	Q99497	*djr-1.1*, *djr-1.2*	[a], [b]
Superoxide dismutase	SOD1	P00441	*sod-1* *, *sod-4* *, *sod-5* *	[a], [b], [20]
	SOD3	P08294	*sod-4* *, *sod-5* *	[a], [b], [20]
Tyrosinase	TYR	P14679	*tyr-1* (and 5 others)	[a], [b]
	TYRP1	P17643	*tyr-5* (and 5 others)	[a], [b]

^a^ Human Genome Organisation (HUGO) Gene Nomenclature Committee, https://www.genenames.org, accessed on 17 March 2024. ^b^ Abbreviations: AOC, amine oxidase copper-containing; cox/ctc cytochrome *c* oxidase subunit; CP, ceruloplasmin; DBH, dopamine β-hydroxylase; djr, DJ1 (=PARK7)-related; ENOX, ecto-NADPH oxidase; ERK, extracellular signal-regulated kinase; HEPH, hephaestin; HEPHL, HEPH-like; LOX, lysyl oxidase; LOXL, LOX-like; MAPK, mitogen-activated protein (MAP) kinase; MAP2K, MAPK kinase; mek, MAPK/ERK kinase; MOXD, monooxygenase, DBH-like; MT-CO, mitochondrially encoded cytochrome *c* oxidase subunit; PAM/pamn, Peptidyl-glycine α-amidating monooxygenase; PARK, parkin; pgal, peptidylglycine α-amidating lyase; pghm, peptidylglycine-α-hydroxylating monooxygenase; semo, SELENBP1 ortholog with methanethiol oxidase activity; SOD, superoxide dismutase; SELENBP, selenium-binding protein; tbh, tyramine β hydroxylase; TYR, tyrosinase; TYRP, TYR-related protein. ^c^ As listed at https://www.uniprot.org (accessed on 17 March 2024). ^d^ All entries denote orthologs by sequence. Proven functional orthologs of the human proteins are additionally marked with an asterisk (*). In the case of Cu enzymes, this means that the same enzymatic activity as that of the human ortholog was demonstrated. ^e^ [a] Ortholog predicted according to OrthoList2 [8]; [b] predicted using the DIOPT (DRSC Integrative Ortholog Prediction Tool, version 9.0) ortholog finder (https://www.flyrnai.org/cgi-bin/DRSC_orthologs.pl (accessed on 17 March 2024); [21]); only genes with a “moderate” or “high” confidence rank were selected. ^f^ Nomenclature used for human and *C. elegans* proteins and genes: (i) Human proteins are in all capitals, without hyphens before numbers; (ii) human genes are all capitals/italics; (iii) *C. elegans* proteins are all capitals, with a number separated by a hyphen; (iv) *C. elegans* genes in lower case/italics, hyphenated. Example: (i) SOD1, (ii) *SOD1*, (iii) SOD-1, (iv) *sod-1*; (i) COX17, (ii) *COX17*, (iii) COX-17, (iv) *cox-17* (for the second example, see Table 2).

A search on UniProt (organism: *C. elegans*, keyword “copper”) for *C. elegans* Cu-binding proteins was performed, followed by a reverse search for human orthologs to these nematode proteins (using the DIOPT ortholog prediction tool referred to in the legend to Table 1). All of the mere 40 entries resulting from the UniProt search or their predicted human orthologs were already listed in Table 1, Table 2 and Table 3. Moreover, we performed a search on the Alliance of Genome Resources page (https://www.alliancegenome.org; keyword “copper ion binding” [22,23], accessed on 11 April 2024) using the AmiGO 2 tool (using the filter “*Caenorhabditis elegans*”; [24]), for *C. elegans* Cu-binding proteins. In summary, the number of known or predicted Cu proteins in *C. elegans* is lower than that in humans.

There are two caveats with these tables: (i) Many of the orthologs were identified solely through predictions based on sequence analyses; whether they are, in fact, functional orthologs is not clear in many cases. As a consequence, all orthologs in Table 1, Table 2 and Table 3 can be considered orthologs by sequence. Some have also been demonstrated to be functional orthologs and are marked with an asterisk in the Tables, as described in the respective table legends. (ii) Even if the functional analogy of a *C. elegans* protein was demonstrated experimentally, it was not always clearly shown that the ortholog is also a Cu-binding protein. With these caveats in mind, it is interesting to note that several proteins share the same ortholog, and this goes both ways: sometimes several human proteins have one common ortholog (e.g., the three human ferroxidases in Table 1) and sometimes a number of *C. elegans* orthologs exists for only one human protein (e.g., tyrosinase, Table 1). This likely reflects two aspects: (i) a diversification of protein isoforms, depending on the biological need, e.g., tissue specificities or the regulation of, and changes in, gene expression in different developmental stages of the organism; (ii) some of the predicted *C. elegans* orthologs will have to be tested for functional homology. An example is listed in Section 3.2.1 below: Of the 10 predicted orthologs of the human copper transporter, CTR1, only one remained on the list, based on experimental evidence.

The remainder of this text will provide a comparison between Cu homeostasis in humans and *C. elegans*. Both differences and similarities will help assess the usefulness of this model organism for answering concrete research questions regarding human Cu homeostasis and discrete aspects of the biological significance of human Cu proteins.

## 3. Copper Homeostasis in *C. elegans*

### 3.1. Copper Uptake and Distribution

In *C. elegans*, Cu (like other metal ions) is usually ingested together with food via the pharynx and enters the intestine for resorption [25,26,27]. Unutilized copper, like other unused nutrients, will likely be excreted by simple intestinal passage through the anus.

Following intestinal resorption, i.e., import into intestinal cells, Cu may be transferred to various intracellular compartments and bound to different proteins for cellular storage or detoxification (see Section 3.2.2). Export from intestinal cells into the pseudocoelomic cavity is required for Cu distribution across the worm, similar to general nutrient exchange and distribution.

**Figure 1:**
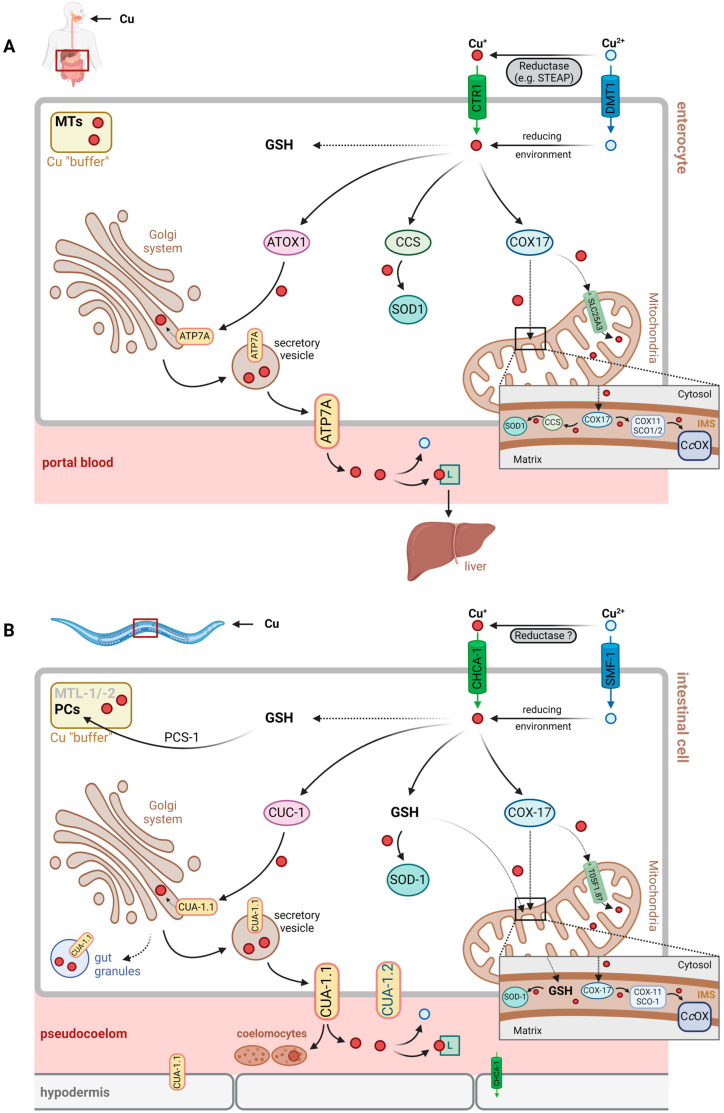
Schematic representation of copper (Cu) uptake and (cellular) distribution in: (**A**) humans and (**B**) *C. elegans*. Orthologs share the same color and style in (**A**,**B**). Following uptake of cupric (Cu^2+^, blue circles) and cuprous (Cu^+^, red circles) ions into cells, the predominantly present reduced form (Cu^+^) is distributed by chaperones or glutathione (GSH): (i) to cytosolic target proteins, such as superoxide dismutases (SODs), (ii) to mitochondria, in order to load cytochrome *c* oxidase (C*c*OX) in the inner mitochondrial membrane and the Cu,Zn-SOD in the intermembrane space (IMS), or (iii) to the Golgi network, in order to load Cu proteins to be secreted. Metallothioneins (MTs) in human cells (**A**), or phytochelatins (PCs) in *C. elegans* (**B**) may buffer excess Cu. *C. elegans* also has metallothioneins (MTLs), but their Cu-binding capability is disputed. PC synthase (PCS) catalyzes the production of PCs from GSH in *C. elegans*. Following export of Cu from cells, both cuprous and cupric ions may be transported/distributed by ligands (Ls). In humans, this may be albumin, among others, for *C. elegans*, the L is unknown. See text for further details. Abbreviations not defined in the captions to Table 1, Table 2 and Table 3: DMT, divalent metal ion transporter; STEAP, six-transmembrane epithelial antigen of the prostate; SMF-1, yeast SMF divalent cation transporter homolog. Figure created with BioRender.com.

It is unclear at present, which Cu-binding factor contributes to distribution via pseudocoelomic fluid, but, similar to the human system [28], histidine or His-containing peptides, as well as general transport proteins that happen to bind Cu, may be assumed to contribute. Cu buffering and detoxification would have to rely on similar transport mechanisms and may be achieved through uptake by coelomocytes, which are capable of endocytosis and the accumulation of macromolecules [29,30] (Figure 1, see also Section 3.2.3.).

Cu resorption by intestinal cells is usually more effective in the presence of food. Accordingly, feeding *Escherichia coli* (*E. coli*) OP50, the standard food source of laboratory *C. elegans*, increases the dose-dependent toxicity of copper compared to copper exposure without food [26]. But when food is available, a higher food-to-copper ratio can attenuate Cu toxicity [31]. Interestingly, the exposure of *C. elegans* to Cu, or feeding bacteria with high Cu content, leads to the increased expression of the metal-responsive gene *numr-1* in the pharynx [27,32], suggesting that the worm’s response to Cu stress is already initiated in the pharynx, where the food is ground prior to passing into the digestive tract. Jackson et al. reported X-ray fluorescence data suggesting a homogeneous distribution of Cu in *C. elegans* following uptake, and they did not see any variation in this pattern independent of whether Cu was supplied with or without food [33]. Based on the observed homogeneous distribution, they also state that, next to ingestion as the likely major path of uptake, diffusion across the cuticle cannot be entirely ruled out [33].

### 3.2. Cellular Cu Import, Distribution and Tolerance

#### 3.2.1. Import

In humans, CTR1 (SLC31A1) is the major copper import protein, allowing for the transport of Cu^+^ across the plasma membrane [1]. The extracellular N-terminal region not only binds Cu^+^, shuttling it to the CTR1 pore for import, but it also binds Cu^2+^, facilitating its reduction prior to import [34], be that through extracellular reductases, such as STEAP family proteins [35], or low molecular mass reductants, such as ascorbate [34].

Yuan et al. identified 10 potential CTR1 orthologs in *C. elegans*, whose amino acid sequence is 30–40% identical to the human protein [36]. Of these, the most relevant was determined as F58G6.9, then named CHCA-1 (CTR1 homolog required for copper accumulation; see Table 2, Figure 1), based upon the findings that: (i) its expression in the intestine and hypodermis during Cu deficiency is enhanced, and (ii) it is required for normal growth, reproduction and Cu accumulation under Cu-deprived conditions [36]. In line with the proposed role of the protein, a *chca-1*-deficient strain had lower Cu levels under Cu-deficient conditions than wild-type worms. Cu supplementation was then demonstrated to normalize Cu content and to rescue the growth defects elicited by Cu deficiency, suggesting that CHCA-1 may not be the only functional Cu importer in intestinal cells [36].

**Table 2:** Human Cu proteins and their *C. elegans* orthologs (II): Cu transporters and Cu chaperones.

HGNC Symbol ^a,b^	UniProt-ID ^c^	*C. elegans* Gene Ortholog ^b,d^	Reference ^e^
ATOX1	O00244	*cuc-1* *	[37,38]
ATP7A	Q04656	*cua-1* *	[39]
ATP7B	P35670		
CCS	O14618	no ortholog	[20]
COMMD1	Q8N668	no ortholog	[a], [b]
COX11	Q9Y6N1	*cox-11*	[a], [b]
COX17	Q14061	*cox-17*	[a], [b]
CUTC	Q9NTM9	*cutc-1*	[40]
SCO1	O75880	*sco-1*	[a], [b]
SCO2	O43819		
SLC25A3	Q00325	T05F1.8 (and 3 others)	[a], [b]
SLC31A1 (CTR1)	O15431	*chca-1* *	[36]
SLC31A2 (CTR2)	O15432		

^a,c,d,e^ See caption to Table 1. ^b^ Abbreviations: ATOX, antioxidant 1 Cu chaperone; ATP7A/B, Cu-transporting ATPase α/β; CCS, Cu chaperone for superoxide dismutase; chca, Ctr1 homolog required for Cu accumulation; COMMD1, Cu metabolism domain containing 1; COX11/17, cytochrome *c* oxidase Cu chaperone COX11/17; CTR, Cu transporter; cua, Cu ATPase; cuc, Cu chaperonin; CUTC, Cu tolerance protein C; SCO, synthesis of cytochrome *c* oxidase; SLC31A1/2, solute carrier family 31, subfamily A, isoform 1/2.

Whereas CTR1 orthologs have been identified in *C. elegans*, no ortholog to STEAP proteins is known. Assuming that CHCA-1, like CTR1, is a Cu^+^-specific transporter, this suggests that either the reduction of divalent Cu prior to uptake is through a different type of reductase or that small reductants, such as ascorbate, contribute to Cu uptake.

In addition to CTR1, the divalent metal ion transporter 1 (DMT1, SLC11A2), known to transport Fe^2+^ as well as other divalent metal ions [41], was proposed as a potential Cu importer in humans [42]. This was challenged by studies testing for the transport of metal ions into *Xenopus* oocytes expressing DMT1, concluding that neither Cu^+^ nor Cu^2+^ was a substrate [43]. Nevertheless, further studies suggest that DMT1 may be used by human cells for Cu import, in addition to CTR1 [44].

Au et al. identified three DMT1 orthologs in *C. elegans*, encoded by *smf-1*, *smf-2* and *smf-3* (named after, and found to be homologs of, the yeast SMF divalent cation transporter), which are associated with the transport of manganese and iron [45]. A study on toxic and neurotoxic effects of Cu and of Cu nanoparticles in *C. elegans* suggests that SMF-1 (but not SMF-2) contributes to Cu uptake, enhancing the Cu-induced stress response and toxicity [46]. Similar to the human system and DMT1, the extent to which SMF proteins contribute to Cu uptake in *C. elegans* is unclear.

#### 3.2.2. Intracellular Cu Distribution

**Figure 2:**
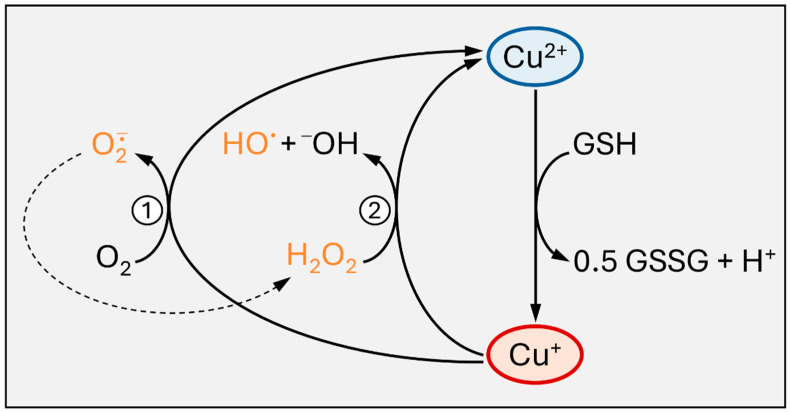
Cu toxicity: generation of reactive oxygen species (ROS). Intracellular reduction of cupric (blue) to cuprous (red) ions, for example by glutathione (GSH), may result in Cu^+^ redox cycling (1), yielding superoxide from molecular oxygen. The resulting Cu^2+^ may again be reduced, restarting the cycle. Superoxide will spontaneously disproportionate to generate oxygen (not shown) and hydrogen peroxide (dashed arrow). (2) In a Fenton-type reaction, Cu^+^ may reduce hydrogen peroxide, resulting in the generation of hydroxyl radicals.

Following import, intracellular Cu^+^ is bound by glutathione (GSH) or by Cu chaperones [47,48]. These thiol-containing chelating peptides/proteins help to avoid undesired redox reactions, such as Cu^+^ redox cycling, generating superoxide and hydrogen peroxide, or the Fenton-type reduction of peroxides by Cu^+^, resulting in hydroxyl radical formation (Figure 2). Moreover, they distribute copper subcellularly, loading Cu-dependent proteins or feeding Cu transporters.

In humans, Cu chaperones are known to bind Cu^+^ after cellular uptake, among them ATOX1 (antioxidant protein 1), CCS (copper chaperone for superoxide dismutase, SOD) and COX17 (cytochrome *c* oxidase copper chaperone) [47]. They allow for Cu distribution in three major directions, i.e., the Golgi system and the secretory pathway, as well as the plasma membrane (ATOX1, delivering Cu^+^ to the Cu-transporting ATPases, ATP7A and ATP7B), cytoplasmic as well as nuclear proteins (CCS, loading Cu,Zn-SOD with Cu) and mitochondria (COX17, providing Cu for subunits of cytochrome *c* oxidase) [47,48].

Orthologous Cu chaperones in *C. elegans* exist (Table 2) and serve the same purpose: preventing Cu-induced oxidative damage and promoting Cu distribution (see Figure 1).

(A) The ATOX1→ATP7A/B Pathway in *C. elegans*: CUC-1→CUA-1

ATOX1 transfers Cu^+^ to membrane-bound Cu(I) exporters, the Cu-ATPases ATP7A and ATP7B, also known as the Menkes disease protein and Wilson’s disease protein, respectively, due to their deficiencies eliciting the respective pathobiochemical alterations in humans. Both proteins are localized to the membrane of the trans-Golgi network and transport Cu^+^ from the cytosol to the lumen of the Golgi compartment, where Cu-dependent proteins destined for secretion, such as ceruloplasmin in hepatocytes, are loaded with Cu. Through vesicular transport, ATP7A/B may also be relocalized to the plasma membrane, supporting Cu export, e.g., under conditions of Cu overload [49]. ATP7A and ATP7B differ with respect to their expression patterns in tissues, with ATP7A being widely expressed, except in the liver, and ATP7B predominantly expressed in liver [49]. They also differ with regard to their subcellular trafficking from the Golgi network to the plasma membrane in polarized epithelial cells exposed to high levels of Cu: whereas ATP7A migrates to the basolateral membrane, ATP7B is found in the apical membrane under such conditions [49,50]. Mutations in the gene encoding ATP7A lead to Menkes disease, which is characterized by a severe systemic copper deficiency, as the export of Cu from enterocytes (basolateral membrane) to portal blood is impaired. Deficiencies in ATP7B cause Wilson’s disease, which is associated with Cu toxicity due to impaired Cu export from the liver and hepatic hyperaccumulation of Cu [47].

CUC-1 (Cu chaperonin 1) is the *C. elegans* ortholog of human ATOX1 and relays copper to CUA-1 (Cu-ATPase 1), the (single) ortholog of both human ATP7A and ATP7B (with 44% amino acid identity and several highly conserved domains) [39].

In adult *C. elegans*, translational [5′-region::ORF fragment::GFP] reporter constructs of both *cuc-1* and *cua-1* are predominantly expressed in the intestine, whereas larvae have the highest expression of both in hypodermal cells [37]. This suggests parallel expression patterns of these genes and is in line with a cooperative role of the corresponding proteins in Cu traffic. One exception regarding parallel expression patterns was noted: *cua-1*, but not *cuc-1*, is also expressed in pharyngeal muscle cells [37,39]; the significance of this finding still needs to be explored.

Deletion of the *cuc-1* gene causes a reduction in worm length, brood size and survival under basal copper availability conditions [38]. However, the size of these effects and the differences between wild-type (WT) worms and *cuc-1*-deficient mutants does not change drastically due to Cu supplementation, implying that the impaired outcomes due to *cuc-1* deletion are not owing to Cu availability per se [38]. Similarly, the expression of *cuc-1* is not altered by the supplementation of WT worms with copper or iron [37]. Rather, the effects on fertility in nematodes lacking *cuc-1* may be a result of the impaired maturation of the gonads, based on the defective migration of distal cells [38].

The activity of CUA-1 as a Cu transporter was suggested by complementation assays in yeast and murine cells: the heterologous expression of *cua-1* was capable of rescuing a deficiency in Ccc2, an ortholog of ATP7A/B in *S. cerevisiae* [51]. Moreover, the transgenic expression of *cua-1.1::gfp* in Atp7a^−/−^ mouse embryonic fibroblasts normalized the Cu concentrations in cells with over-accumulated Cu, implying that CUA-1.1::GFP contributes to Cu export [39]. Recently, Catalano et al. generated a novel *cua-1* mutant strain, with CUA-1 altered in a way to imitate a common ATP7B variant present in Wilson’s disease patients. These *cua-1* mutant worms were considerably less resistant to Cu than wild-type worms, as evident from data showing delayed development, decreased life span and neuronal damage [52], rendering this *C. elegans* strain a suitable model for the analysis of Cu homeostasis and toxicity in Wilson’s disease.

In *C. elegans*, the *cua-1* transcript has two splice isoforms, *cua-1.1* and *cua-1.2*. The latter differs from *cua-1.1* in that it is devoid of the first two exons that encode one of the putative N-terminal metal-binding domains [39].

**Figure 3:**
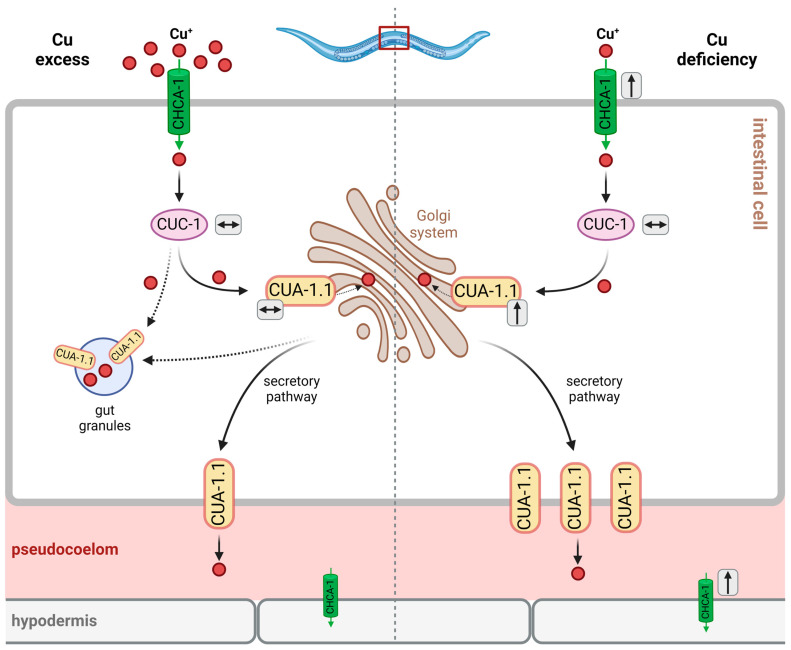
Schematic representation of cellular processes in *C. elegans* upon exposure to excess Cu (left panel) and under conditions of Cu deprivation (right panel). Upon copper overload, CUA-1.1 localizes to gut granules (see Section 3.2.3). To ensure availability and distribution of Cu, even under Cu deprivation, *chca-1* expression is upregulated in the intestine and hypodermis and CUA-1.1 localizes to the intestinal basolateral membrane. Black arrows in grey boxes indicate “upregulation” and “no change” in protein expression, respectively, after Cu excess or Cu deprivation. Figure created with BioRender.com.

Similar to ATP7A/B in humans, the localization of CUA-1.1 in intestinal cells varies with copper availability (see Figure 3): under basal conditions, CUA-1.1 localizes to basolateral membranes and intracellular compartments, such as the Golgi apparatus; under conditions of Cu deficiency, *cua-1* expression is upregulated, and CUA-1.1 predominantly localizes to basolateral membranes, which is required for the efflux of copper into the pseudocoelom, allowing for Cu distribution to peripheral tissues [39]. Cu overload causes the relocalization of CUA-1.1 to the membrane of gut granules in the intestinal cells of *C. elegans*, allowing for Cu sequestration (rather than the distribution of toxic levels to peripheral tissues) [39]. In hypodermal cells, CUA-1.1 is localized to the plasma membrane independently of copper supply. The expression of *cua-1*, however, is increased by Cu deprivation in L1 worms, as demonstrated both by the decreased endogenous *cua-1* mRNA levels and the use of a translational reporter [39] (see Figure 3). The second isoform, CUA-1.2, is constitutively localized to the basolateral membrane of intestinal cells [39]. Chun et al. hypothesized that intestinal CUA-1 may serve both functions that, in humans, are attributed to intestinal ATP7A and hepatic ATP7B. The authors investigated the effects of intestine-specific downregulation of *cua-1.* The effects—a reduction in fecundity under basal and copper-depleted conditions, with progeny numbers comparable to whole-body *cua-1* RNAi—suggest that the regulation of intestinal *cua-1* expression is the key to regulating Cu uptake and toxicity [39]. In fact, RNA interference targeting *cua-1* or *cuc-1*, following Cu supplementation, leads to an approximate 30% reduction in the total copper content compared to the controls [39].

(B) Glutathione-Dependent, but CCS-Independent, Cu Transfer to SODs in *C. elegans*

Superoxide dismutases (SODs) catalyze the disproportionation (dismutation) of metabolically generated superoxide to H_2_O_2_ and molecular oxygen, thereby contributing to antioxidant defense in combination with peroxidases that catalyze the removal of H_2_O_2_ [2]. Humans have three SODs, SOD1-SOD3. SOD1 is a cytosolic enzyme that is also present in the mitochondrial intermembrane space (IMS) [53,54] and that clearly requires Cu (as well as Zn) for full activity. When the protein now called SOD1 was first identified as an enzyme with SOD activity in 1969, it was already known that it contains Cu [55], and crystal structure analyses were published in the early 1980s [56]. Similarly, extracellular SOD (EC-SOD, SOD3) is a Cu/Zn-containing SOD, albeit structurally distinct from SOD1. Lastly, SOD2 is an enzyme in the mitochondrial matrix, with manganese at its active site (Mn-SOD).

*C. elegans* has five SOD orthologs [20,57,58]. Two cytosolic forms (analogous to human SOD1) are encoded by *sod-1* and *sod-5*, whereas *sod-4* encodes an extracellular SOD (analogous to human SOD3). All three are Cu- and Zn-dependent SODs. Mitochondrial SODs (analogous to human SOD2) are encoded by *sod-2* and *sod-3*. The gene encoding Cu-dependent cytosolic SOD-5 is expressed mainly in the dauer larva stage, while *sod-1* encodes the major Cu,Zn-SOD isoform in general [20,57]. The expression patterns observed for an *sod-5::gfp* reporter suggest that *sod-5* is expressed in a few neurons of the amphid, a chemosensory organ, including ASI and ASK neurons [57], which are known to contribute to Cu sensing [59,60].

The enzymatic activity of these SODs requires Cu at the active site, which cycles between its cuprous and cupric states. Human SOD1 is loaded with Cu by CCS, also in the mitochondrial IMS [54], a pathway also found in other eukaryotic organisms (for review, see [61]). In humans, CCS-dependent Cu transfer to SOD1 is, to a minor extent, complemented by CCS-independent mechanisms. These, however, have not been entirely elucidated with respect to the molecule(s) transferring Cu to SOD; several thiol-containing molecules have been hypothesized, including glutathione (GSH) [61].

In *C. elegans*, there is no CCS ortholog. A series of experiments in yeast with heterologously expressed human SOD1 and worm SOD-1 demonstrated that the protein from *C. elegans* required GSH and was inactive without GSH even in the presence of (yeast) CCS [20]. GSH, therefore, appears to “replace” CCS in *C. elegans*. Or, in other words, although *C. elegans* cannot be used to investigate the physiological effects of CCS, it may still serve as a model for CCS-independent Cu transfer, which was also observed in human cells (see above).

Interestingly, it was suggested that Cu may be transferred to proteins other than SODs by GSH and also glutaredoxins: isolated human GRX1 was demonstrated to transfer Cu to ATOX1 or ATP7B [62]. Considering the similarities in the glutaredoxin systems between humans and *C. elegans* [63,64], the role of glutaredoxins in Cu homeostasis may be explored further using *C. elegans* as an appropriate model.

(C) Mitochondrial Cu Chaperones

The mitochondrial respiratory chain requires Cu, as complex IV, i.e., cytochrome *c* oxidase (C*c*Ox), is a cuproenzyme complex. Of its two Cu sites, Cu_A_ and Cu_B_, Cu_A_ is a dicopper site. Another mitochondrial copper protein frequently overlooked as it is usually regarded predominantly as cytoplasmic, is SOD1 (see above), which is also present in the mitochondrial IMS, as is CCS, the chaperone required for the Cu loading of SOD1 [54]. Both SOD1 and CCS, however, are proposed to enter the IMS in an immature state, not carrying Cu [54,65]; hence, the Cu for loading SOD1 needs to be from a different source. Whereas the transportation of Cu into the mammalian mitochondrial matrix is catalyzed by the mitochondrial phosphate carrier, SLC25A3 [12], it is unclear how Cu reaches the IMS. Several hypotheses were discussed by Zischka and Einer [66], including glutathione-mediated transportation from the cytosol to the IMS and Cu redistribution between the matrix, IMS and cytosol by an unidentified ligand.

Redistribution of Cu from the mitochondrial matrix to the IMS has been suggested by experiments with murine cells deficient in the SLC25A3 ortholog and displaying lower C*c*Ox activity: overexpression of a SLC25A3 mutant capable of transporting Cu, but not phosphate, rescued not only the mitochondrial Cu levels but also the C*c*Ox activity [67]. In line with these findings, SOD1 (but not SOD2) activity is lowered in the mitochondria of SLC25A3-deficient cells [12].

Cu is provided to C*c*Ox by a “chaperone relay” in the IMS, starting with COX17, which further distributes Cu to SCO1/SCO2 and COX11, which then donate their Cu to the C*c*Ox Cu_A_ and Cu_B_ sites, respectively [66,68].

It is likely that *C. elegans* has an ortholog of the human mitochondrial phosphate and Cu carrier SLC25A3, as a study on the evolutionary development of expressing two separate (as in yeast) vs. one (as in mammals) mitochondrial carrier for phosphate and/or Cu suggests [67]. However, no functional analysis has been conducted in *C. elegans* to that effect; OrthoList [8] and WormBase list more than one ortholog, including T05F1.8 (see Table 2). Future studies will have to show which of these contributes to Cu transportation across mitochondrial membranes. Similarly, not much information on the functional aspects of mitochondrial copper chaperones is available for *C. elegans*. Again, a search of OrthoList [8] yields merely predicted orthologs of the genes encoding these chaperones (*cox-17*, *cox-11*, *sco-1*), and analyses of the expression levels in response to Cu exposure of worms of some of these genes have been reported, such as, very recently, *cox-17* [69].

(D) CUTC-1—A Chaperone?

In search of orthologs to bacterial Cu tolerance factors, including CutC [70], human CUTC1 was identified as a ubiquitously expressed cytoplasmic (and, in part, nuclear) protein of 29 kDa likely involved in Cu homeostasis [71]. Analysis of Cu binding by CUTC1 and of structural features revealed that it is present as a tetramer in human cells and it does not have a classic high-affinity binding site present in Cu chaperones or transporters, but that it binds one Cu^+^ per monomer, comparatively weakly, with a dissociation constant in the micromolar region [72]. It is, thus, unlikely that CUTC1 is a Cu chaperone or involved in Cu trafficking. The depletion of CUTC1 by siRNA caused increased Cu sensitivity and apoptosis in HepG2 human hepatoma cells, while not altering the overall Cu levels in cells [73].

The *C. elegans* ortholog, CUTC-1, encoded by *cutc-1*, shares a high degree of identity (39%) and similarity (59%) with the human protein, and is also likely a cytoplasmic protein [40]. Similar to the human protein, it does not contain the usual Cu-binding motifs; nevertheless, RNA interference downregulating the expression of *cutc-1*, while not affecting overall Cu content, caused an exacerbation of the Cu-induced effects, such as the development of egl (egg-laying defective) and pvl (protruding vulva) phenotypes [40]. At higher Cu concentrations, the RNAi of *cutc-1* also lowers the brood size and worm length [40].

These findings, together with the additional information that *cutc-1* expression is reduced upon copper overload [40], suggest that CUTC-1, like the human counterpart, CUTC1, (i) is neither an importer or exporter protein (overall cellular Cu levels are not affected by its absence), (ii) nor a classic Cu chaperone (weak binding of Cu) or (iii) a Cu buffer (as its expression is downregulated, rather than upregulated, upon Cu stress). All available evidence, however, indicates that it is involved in Cu homeostasis. The exact mode of its involvement needs to be defined and, owing to the listed similarities between the human and worm protein, *C. elegans* seems to be a good model to further investigate the significance of human CUTC1 and worm CUTC-1.

#### 3.2.3. Cellular Cu Buffers/Cu Tolerance

Intracellular Cu buffers contribute to the Cu tolerance of *C. elegans* by preventing undesired effects of excess Cu ions, including the generation of ROS and inhibition of proteins by unspecific interactions (see above).

**Table 3:** Human Cu proteins and their *C. elegans* orthologs (III): Cu buffering proteins and other Cu-binding proteins, including those with unclear functions.

HGNC Symbol ^a,b^	UniProt-ID ^c^	*C. elegans* Gene Ortholog ^b,d^	Reference ^e^
AFP	P02771	no ortholog	[a], [b]
ALB	P02768	no ortholog	[a], [b]
AHCY	P23526	*ahcy-1*	[a], [b]
APP	P05067	*apl-1* *	[14,74]
APLP2	Q06481		
CUTA	O60888	F35G12.7	[a], [b]
F5	P12259	*ddr-1*	[b]
GPC1	P35052	*gpn-1*	[a], [b]
LTF	P02788	no ortholog	[a], [b]
MEMO1	Q9Y316	*memo-1* *	[75]
MT1 (9 isoforms)	various	*mtl-1/mtl-2* *	[76,77]
MT2A	P02795		
MT3	P25713		
MT4	P47944		
MUC2	Q02817	T01D3.6	[b]
MUC5AC	P98088	Y69H2.10 (and 3 others)	[b]
MUC5B	Q9HC84	Y69H2.10 (and 7 others)	[b]
PRNP	P04156	no ortholog	[a], [b]
S100A12	P80511	no ortholog	[a], [b]
S100A13	Q99584		
S100A5	P33763		
S100B	P04271		
SNCA	P37840	no ortholog	[a], [b]
SPARC	P09486	*ost-1*	[a], [b]

^a,c,d,e^ See caption to Table 1. ^b^ Abbreviations: AFP, α-fetoprotein; ALB, albumin; AHCY, adenosylhomocysteinase; apl, amyloid precursor-like; APLP2, amyloid-β-precursor-like protein; APP, amyloid-β precursor protein; CUTA, cutA divalent cation tolerance homolog; ddr, discoidin domain receptor; F5, coagulation factor V; GPC/gpn, glypican; LTF, lactotransferrin; MEMO, mediator of cell motility; MT/mtl, metallothionein; MUC, mucin; ost, osteonectin (SPARC)-related; PRNP, prion protein; S100, S100 calcium-binding protein family; SNCA, α-synuclein; SPARC, secreted protein acidic and rich in cysteine.

(A) Sequestration by Gut Granules

Owing to findings on CUA-1.1 being relocated to gut granules upon Cu overload [39], these lysosome-like organelles were hypothesized to contribute to Cu tolerance through the sequestration of excess Cu (see Figure 3).

Studies on mutant strains with defective gut granule formation show that, while these organelles may contribute to Zn storage and accumulation, as inferred from alterations in overall worm Zn content, both with and without Zn supplementation, no such effect was observed for Cu. The Cu content under basal conditions (i.e., without Cu supplementation) was unaltered, independent of gut granule formation [78]. Moreover, the authors found that worms defective in gut granule biogenesis “were similar to wild-type animals in sensitivity to dietary (…) copper” [78]. A later study focusing on CUA-1 and gut granules, however, investigated the response to the exposure to elevated Cu levels and found that both the total Cu levels and Cu sensitivity were indeed altered in worms defective in gut granule biogenesis relative to wild-type controls: Cu accumulation under both basal and Cu-supplemented conditions was attenuated, and sensitivity to high Cu levels was increased in these worms [39].

The discrepancies between these two studies may result from different Cu exposure conditions and the different general focus of the studies; in summary, it appears that gut granules, while they are no Cu storage vesicles under basal conditions, may serve as subcellular Cu sequestration compartments, contributing to Cu detoxification under conditions of exposure to excess Cu.

(B) Thiol-Mediated Chelation of Cu: Metallothioneins, Phytochelatins, Glutathione

Metallothioneins (MTs) are small cysteine-rich proteins binding metal ions, including Zn^2+^, Cd^2+^ and Cu^+^, through interaction with thiol moieties; human MT1a, for example, consists of 61 amino acyl residues, 20 of which are cysteines (for a thorough review on MTs, see [79]). Formally, the (apo-) protein is thionein, which becomes a metallo-thionein only upon binding metal ions; nevertheless, this distinction is usually no longer made when discussing the biosynthesis of MTs or the expression of genes encoding MTs.

MTs strongly bind Cu^+^ [79] and may chelate excess Cu ions not bound by chaperones or Cu proteins. This Cu-binding capacity of metallothioneins is even exploited therapeutically in the treatment of Wilson’s disease [80]: oral application of high doses of Zn^2+^ causes the upregulation of metallothionein formation in enterocytes through the activation of metal regulatory transcription factor 1 (MTF1) [81]. Cu is then bound by enterocytic MT and not passed on to portal blood; rather, it is excreted with the natural shedding of enterocytes.

There is no ortholog of MTF1 in *C. elegans*, but *mtl-1* and *mtl-2* were identified as genes encoding metallothioneins, MTL-1 and MTL-2 (Table 3), which share the typical properties of metallothioneins, such as their small size (consisting of 75 and 63 amino acids, respectively), their high cysteine content (25% and 29%, respectively) and the presence of no more than one aromatic amino acid [82,83]. It is interesting to note that, while upregulated by Cd^2+^ exposure [83], the expression of *mtl-1*/*mtl-2* in *C. elegans* tends to be downregulated under Cu stress [69,84], which renders it unlikely that MTL-1/2 serve as major Cu buffers. In line with this, Cu-binding activity was demonstrated to be lower (relative to human MT, and relative to divalent Cd and Zn ions) in *C. elegans* metallothioneins, likely owing to the presence of several histidine residues [85]. Of note, despite these findings, an MTL-2 deficiency was reported to render *C. elegans* sensitive to Cu exposure [86], and a transgenic worm strain expressing *pmtl-2::GFP*, i.e., a GFP reporter coupled to an *mtl-2* promotor fragment, similar to an earlier precursor thereof with a lacZ reporter [87], was successfully used as an indicator strain for subtoxic environmental concentrations of metal ions, including Cu [88].

The tripeptide glutathione (GSH, γGlu–Cys–Gly) was described as forming Cu(I) complexes that may serve as intracellular “Cu transfer hubs”, distributing Cu to Cu proteins or MTs in human hepatoma cells [89], and which can be used to reconstitute Cu-dependent SOD1 [90,91]. GSH was already mentioned above as being capable of transferring Cu to *C. elegans* SOD-1 [20]. Different from MTs, GSH is unlikely to serve as a major buffer molecule to prevent adverse effects of Cu excess by mere chelation and storage; rather it is crucial for the transfer of Cu to buffering molecules, such as MTs, and as a major electron donor for intracellular antioxidant systems, such as glutathione peroxidases that may catalyze the reduction of peroxides generated in the presence of redox-active Cu ions [89]. A role of GSH as being involved in intracellular Cu distribution was also suggested by studies on the effects of GSH depletion on Cu homeostasis in neuronal development in *Drosophila melanogaster* [92]. It is interesting to note that GSH in *C. elegans* can be depleted to some extent in worms, resulting not only in unimpaired, but rather improved survival under (oxidative) stress [93]. It is unclear at present, whether there is a link between this effect and Cu homeostasis, but the expression of genes encoding antioxidant enzymes was upregulated in worms following moderate (but not stronger) GSH depletion [93]. This moderate depletion, while upholding sufficient GSH levels to acutely deal with stressful stimuli, may serve as a signal for cells to prepare for oxidant challenges as they occur, for example, upon exposure to redox-active metal ions.

Next to serving as a major cellular antioxidant and as a Cu distributor, GSH has a peculiar (as originally thought to be plant- and microbe-specific) third role in Cu homeostasis in *C. elegans*: it provides building blocks for the biosynthesis of major Cu chelators, phytochelatins. *C. elegans* has a phytochelatin synthase (PCS-1), catalyzing the biosynthesis of phytochelatins, cysteine-rich peptides, from GSH [94,95], by forming polymers of γGlu–Cys (with up to 11 repeats) [96]:

(a) 2 GSH → (γGlu–Cys)_2_–Gly + Gly

(b) (γGlu–Cys)_2_–Gly + γGlu–Cys–Gly → (γGlu–Cys)_3_–Gly + Gly, etc.

In line with the metal-chelating activity of phytochelatins, PCS-1 is required for Cu tolerance in *C. elegans* [97]. Summing up the above evidence on MTs, GSH and phytochelatins, it appears likely that the latter are predominant Cu buffers. A study on Cd tolerance in *C. elegans* has demonstrated the same for Cd resistance, and the authors conclude that their study “showed that metallothioneins do help protect against cadmium toxicity, but not to the same extent as phytochelatins” [98].

A GFP reporter under the control of a *pcs-1* promotor (*ppcs-1::GFP*) is expressed in several tissues, including the hypodermis, the pharynx (grinder, pharyngeal–intestinal valve), the body wall and vulval muscles, and coelomocytes [97].

(C) Coelomocytes Contribute to Cu Tolerance in *C. elegans*

Adult *C. elegans* hermaphrodites have three pairs of coelomocytes, which are located in the pseudocoelom, and were described by Fares and Greenwald as “scavenger cells that continuously and nonspecifically endocytose fluid from the pseudocoelom” [29]. In addition to expressing *pcs-1*, they also express *hmt-1*, a gene encoding an ortholog to the human ATP-binding cassette transporter ABCB6, heavy metal tolerance factor 1 (HMT-1), contributing to Cu uptake into cells, including coelomocytes [97]. Based on these data and on their finding that coelomocyte-deficient nematodes are more sensitive to copper toxicity than WT controls, Schwartz et al. propose that coelomocytes are major contributors to Cu (and Cd, as well as As) tolerance in *C. elegans* [97].

It is unclear at present, in what form Cu enters coelomocytes: is it an unspecific endocytotic uptake of whatever comes along, including Cu complexes, or is it rather a more specific uptake of certain Cu chelates, or even of Cu ions? More recent studies on HMT-1 in *C. elegans* intestinal cells demonstrate that, subcellularly, HMT-1 is linked to the recycling endosomal compartment [99], which may point to either of the first two options, i.e., the endocytotic uptake of Cu complexes.

Another open question concerns the fate of Cu taken up by coelomocytes, and further studies will have to elucidate whether Cu is merely deposited in these cells in an “inert” form, for example as a Cu–phytochelatin complex, or rather stored transiently to serve as a backup system in times of Cu deprivation.

### 3.3. From Orthologs to Conserved Mechanisms: Molecular Aspects of Intracellular Cu Transport

A number of functional and sequence orthologs of Cu proteins have been described above and listed in Table 1, Table 2 and Table 3. Here, we ask to what extent the functional homologies are reflected at a molecular level and, as an example, focus on the well-described “Cu-relay” in human cells from the uptake by CTR1 to the secretory pathway via ATOX1 and the passage of ATP7A/B into the Golgi lumen (for review, see [100,101]).

#### 3.3.1. Conservation of Domains Involved in Cu Uptake by CTR1 and CHCA-1

**Figure 4:**
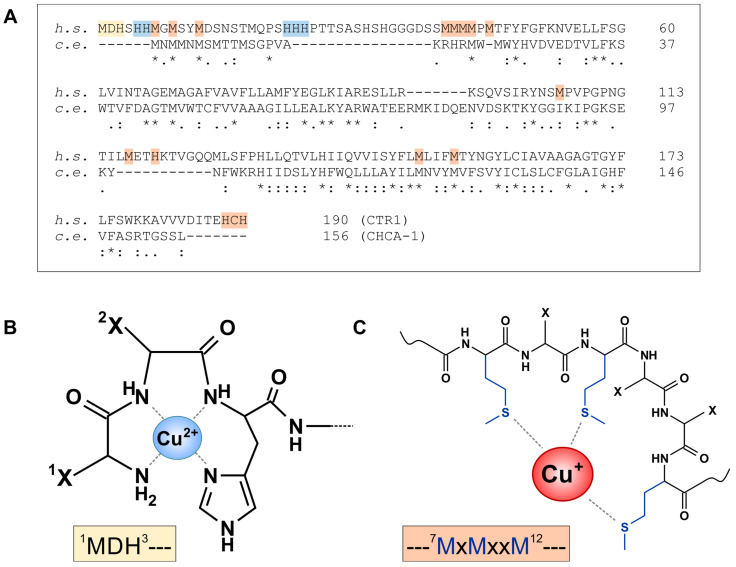
Conserved domains in the copper transporters CTR1 and CHCA-1. (**A**) Sequence alignment of human (h. s.) CTR1 and *C. elegans* (c. e.) CHCA-1, using UniProt entries O15431 and Q5CZ44, respectively, and Clustal Omega alignment tool. Yellow: ATCUN motif; blue: Cu^2+^-binding motif; orange: Cu^+^-binding motif. “*”, “:”, “.” indicate identical, strongly similar and weakly similar residues, respectively. (**B**) Structure of cupric ions bound to the “amino-terminal Cu(II) and Ni(II) binding” (ATCUN) motif in CTR1. Met and Asp side chains are indicated by X. (**C**) Binding of cuprous ions by a methionine-rich motif in the extracellular region of CTR1 [104].

As described above, cellular Cu uptake is predominantly through CTR1, and in the cuprous form. CTR1 is a homotrimeric transmembrane protein of 21 kDa/subunit. Its extracellular N-terminal region binds both Cu^+^ and Cu^2+^, the latter likely to facilitate its reduction prior to relaying it to the transmembrane channel [34]. Several Cu-binding motifs were identified in the N-terminal region (Figure 4A), including two Cu^2+^ sites, an “amino-terminal Cu(II) and Ni(II) binding” (ATCUN) motif, ^1^MDH^3^ [102] (Figure 4B), as well as ^22^HHH^24^ [103]. Three Cu^+^ binding sites per monomer were identified as ^5^HHM^7^/^7^MxMxxM^12^ (with x being any residue) (Figure 4C), ^40^MMM^42^ and ^42^MMxM^45^ [102,103].

The attraction of Cu to the extracellular CTR1 region may be supported by the presence of negative charges, as provided by Asp and Glu residues [105]: The 65 N-terminal residues contain four charged residues, 3 Asp, 1 Glu and 1 Lys, yielding a net negative charge of −3 per monomer.

The predicted extracellular N-terminal region of CHCA-1 is from Met1 to Ala43 (AlphaFold [106]; https://alphafold.ebi.ac.uk/entry/Q5CZ44, accessed 17 March 2024), and this region shares many of the features of CTR1. (i) The N-terminus is rich in methionines (eight Met overall in the extracellular region), providing multiple putative Cu^+^-binding sites: ^1^MxMMxMxMxxM^11^ and ^21^MxMxxH^26^; (ii) although no ATCUN motif is present in the *C. elegans* protein, ^26^HxDxED^31^ could represent a potential Cu^2+^-binding site. As for the cation-attracting charge, there is, however, no net surplus of negative charge (with two Lys, two Arg, three Asp, one Glu, the basic and acidic residues are balanced); nevertheless, it appears that Asp42 is rather close to the putative channel entry and might serve the purpose of attracting Cu ions.

In the CTR1 transmembrane channel, formed by the three monomers, four “methionine triads” are established that may serve as (transiently) Cu^+^-binding rings. Each of these consists of three adjoining methionines provided by the three monomers: M45, M43, M154 and M150; the M45 and M43 triads may be considered entry points into the channel [105]. M150 and M154 are conserved in CHCA-1 (M123, M127), and two further methionines are found in the putative transmembrane region (M46, M74).

The 15 cytoplasmic C-terminal amino acids of CTR1 end in ^188^HCH^190^. From their experiments with C-terminally truncated CTR1, Maryon et al. concluded that the intracellular tail (and the C-terminal HCH) “may act as a barrier, regulating or limiting the rate of copper exit from the pore” [107]. Indeed, the terminal HCH was demonstrated as a Cu^+^-binding site and essential for the intracellular transfer of Cu^+^ to ATOX1 [108]. CHCA-1 does not have a similar site [36]. This could suggest that Cu uptake via CHCA-1 in *C. elegans* is not attenuated by the cytoplasmic tail. Such an unhindered uptake of Cu could mean a growth benefit for *C. elegans*, as worms might be more dependent on quick and efficient Cu import.

#### 3.3.2. Cu Binding by ATOX1/CUC-1 during Transfer to ATP7B/CUA-1

**Figure 5:**
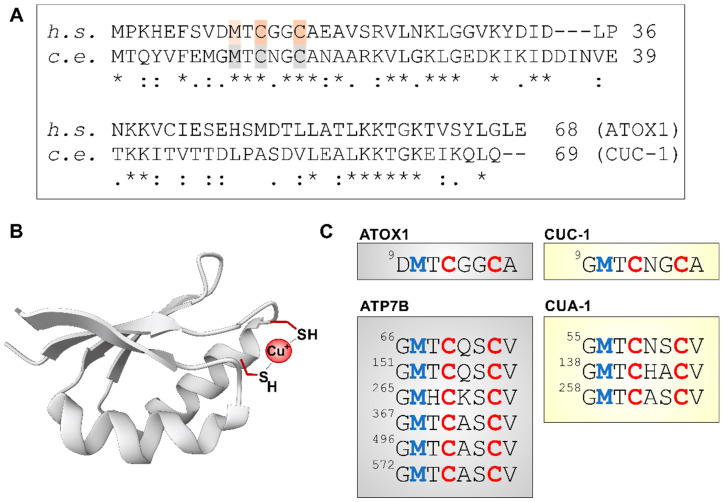
Conserved domains in the ATOX1/ATP7B and CUC-1/CUA-1 pathways. (**A**) Sequence alignment of human (h. s.) ATOX1 and *C. elegans* (c. e.) CUC-1, using UniProt entries O00244 and G5EE41, respectively, and Clustal Omega alignment tool. “*”, “:”, “.” indicate identical, strongly similar and weakly similar residues, respectively. (**B**) The 3D structure of ATOX1 (protein database, PDB ID: 1TL4 [112]), with cysteines 12 and 15 chelating Cu^+^. (**C**) Conserved MxCxxC motif (blue: methionines, red: cysteines) in human (gray) and *C. elegans* (yellow) proteins. In addition to ATOX1 and CUC-1 (see also (**A**)), this motif is conserved in the six N-terminal metal-binding domains of human ATP7B (UniProt P35670) [113] and *C. elegans* CUA-1 (UniProt G5EE14).

After the release of imported Cu^+^ from the CTR1 C-terminus, Cu is bound by ATOX1 through cysteines in a CXXC motif (Figure 5) [109]. In *C. elegans*, CUC-1 shares this motif [37], suggesting that it is not merely an ortholog by sequence (with 41% identity), but is also functionally similar. ATOX1 then supplies ATP7A/B with Cu. ATP7A/B has six copper binding domains (or metal-binding domains, MDB), each with a CXXC motif (Figure 5). The transfer of Cu to MDB4 of ATP7B requires Lys60 of ATOX1 [110,111], which is also conserved in CUC-1 (Lys63, Figure 5A).

It appears that ATOX1 may interact with all six MDBs, but more strongly interacts with MDBs 1, 2 and 4, which then further relay Cu, ultimately, to MDB6 [113,114,115,116]. MDB6 is close to the transmembrane region of the transporter and provides Cu for transmembrane transfer [117]. The *C. elegans* ortholog of ATP7A/B, CUA-1 (44% identity), shares the same copper-binding motifs, but appears to have only three N-terminal MDBs (Figure 5C). Cu binding by ATOX1 requires the cysteines in the CXXC motif to be in their reduced state, which is upheld by GSH and may be facilitated by glutaredoxin GRX1 [109,118].

### 3.4. Regulatory Aspects of Cu Homeostasis in C. elegans: From Altered Gene Expression to Stress Response

Cu homeostasis in *C. elegans* is tightly controlled, as evident from the fact that there is no direct translation of changes in environmental Cu to altered Cu content in worms. For example, a 15-fold increase in the environmental Cu concentration resulted in only a 5-fold increase in Cu content [39]. Up- and downregulation, as well as altered localization of factors involved in Cu homeostasis (which were introduced above), is an essential part of Cu homeostatic processes, including Cu tolerance (see Figure 3). For example, Cu deficiency leads to the increased expression of *chca-1* in the intestine and hypodermis [36] and a moderate upregulation of *cua-1* expression, whereas the expression of *cuc-1* is not changed [38]. Interestingly, excess copper does not alter *cua-1* and *cuc-1* expression [38,39], suggesting that *C. elegans* is rather equipped to secure Cu availability, dealing with the odd exposure to Cu excess by providing Cu buffers, such as phytochelatins (see above).

The tight control of Cu homeostasis is detectable also at the level of gene expression, with patterns of genes expressed changing with the life stage (e.g., L1 vs. L4) and in relation to the Cu concentrations worms are being exposed to [119]. Several genes encoding enzymes crucial to central metabolic pathways are among the responders to an exposure to elevated Cu, including a number of genes involved in fatty acid metabolism. In fact, Cu supplementation resulted in an increase in the lipid content and the size of lipid droplets [119].

Prior to modulating gene expression, signaling cascades are being affected by Cu exposure, including the *C. elegans* ortholog of the human forkhead box transcription factor FOXO3, DAF-16: Similar to Cu stimulating insulin-like signaling in human cells, resulting in an inactivation and nuclear exclusion of FOXO transcription factors [120,121,122], subcellular localization of a DAF-16::GFP fusion protein in *C. elegans* exposed to Cu was also changed, suggesting its inactivation [3,121].

The lack of an MTF1 ortholog in *C. elegans* was already referred to above, which leaves the question open as to which transcriptional regulator (and regulatory upstream pathway) may contribute instead. Interestingly, MDT-15, the *C. elegans* ortholog of human MED15, the human mediator of RNA polymerase II transcription complex subunit 15, is required for upregulation of the expression of the metallothionein genes *mtl-1* and *mtl-2* in response to cadmium or zinc exposure [123]. Whether the same applies for Cu was not tested, but a link to Cu homeostasis exists through the involvement of MDT-15 in regulating the expression of a Cu protein. MDT-15 was demonstrated to be a regulator of the expression of *semo-1* (Y37A1B.5, [124]), encoding SEMO-1, which was demonstrated to be a functional ortholog of the human selenium-binding protein (SELENBP1) [19], and which was demonstrated to be a novel Cu-dependent enzyme [11].

SEMO-1 is an example of an enzyme whose activity is dependent on Cu availability to *C. elegans*: copper supplementation increases the enzymatic activity, whereas it is attenuated by the addition of a copper chelator [11]. The loss of this enzymatic activity in worms grown in the presence of a Cu chelator points to an interesting aspect, namely that while a Cu excess can be buffered to some extent (see above), a Cu deficiency in worms is rather simple to elicit experimentally, which further explains the upregulation of Cu transporters under such conditions, as there appears to be the need for *C. elegans* to be well equipped to uphold Cu levels to some degree in a Cu-deficient environment.

SEMO-1 offers an interesting (but hypothetical) aspect on a potential role of Cu in signaling: the enzymatic activity of the protein is that of a methanethiol oxidase (MTO), catalyzing the oxidation of methanethiol, a breakdown product of methionine, to generate hydrogen peroxide, hydrogen sulfide and formaldehyde [125,126]. Whereas formaldehyde may feed into C1 metabolism, the two other products are well-known redox signaling molecules [127,128]. It remains to be seen whether the link between Cu availability and the SEMO-1-dependent production of signaling molecules translates to a modulation of gene expression and stress resistance of *C. elegans*. In that context, it was demonstrated that the downregulation of *semo-1* expression by RNA interference causes an increased expression of genes encoding antioxidant proteins, including *sod-1* and *sod-2* or all five glutaredoxin genes [129]. An indication that SEMO-1 is indeed involved in the regulation of stress resistance comes from the first experiments with SEMO-1-deficient worms, demonstrating that SEMO-1 may confer resistance against selenite, but at the same time sensitize worms to oxidative stress [19]. It is, however, not known, whether these effects rely on the enzymatic activity of the protein and on the presence of Cu.

## 4. Closing Remarks

Copper is essential to central metabolic pathways in eukaryotic cells. With the need for Cu supply, uptake and organismal as well as the cellular distribution of Cu, comes the need to adapt to changing Cu availability in the environment. This includes both Cu deficiency and Cu excess, as both may have adverse outcomes for the organism. The purpose of this article was to provide a background with respect to whether, and to what extent, *C. elegans* can be useful as an in vivo model for research into mammalian Cu homeostasis.

There are clearly aspects of worm biology that do not allow for drawing direct conclusions regarding the human system. Some examples, in part already mentioned, are the following: (i) At the cellular level, the role of phytochelatins (and of GSH, as it provides the building blocks for these chelating molecules) in Cu buffering is, at present, rather unique among the frequently used multicellular animal models (see Section 3.2.3), as no phytochelatin synthesis has been demonstrated in animals other than nematodes so far. (ii) Some human proteins involved in Cu homeostasis have no orthologs in *C. elegans*, including CCS (see Section 3.2.2) or the metal-responsive transcriptional regulator MTF1 (see Section 3.2.3). (iii) In humans and other vertebrates, the excretion of Cu is, in large part, via feces [130]; it is unclear whether specific Cu excretion occurs in *C. elegans*, and, if it does, how exactly it is achieved.

With such obvious differences in mind, why should *C. elegans* be used as a model system to analyze Cu homeostasis, if, for example, cultured human cells, which do not have an issue with missing orthologs, may be similarly accessible? While questions regarding Cu binding and the enzymatic activity of proteins can certainly be approached at a cellular and even cell-free level, the biological significance of proteins sometimes comes to light in a tissue context only. For example, the above-referenced SEMO-1, as well as the human ortholog, SELENBP1, was demonstrated to be a Cu-dependent enzyme [11], but the significance of its methanethiol oxidase activity and the products generated or the substrate being depleted has not been fully elucidated. From the data on *C. elegans* (but not from human cultured cells), it is clear that SEMO-1 is involved in the regulation of stress resistance and lifespan [19,124]. Although the basis for this effect, and to what extent transcellular and paracrine effects play a role, remains to be resolved, it provides a read-out for the biological activity of the protein beyond its enzymatic activity (whose significance is unknown). *C. elegans*, therefore, provides an accessible model to investigate the biological significance of proteins beyond the cellular level. The results may be pointers for further research using mammalian cells and animal models.

This also applies to the study of Cu homeostasis, for which *C. elegans* is an excellent model for several reasons. (i) A multitude of human proteins involved in Cu homeostasis have structural and functional orthologs in *C. elegans*, as listed in Table 1, Table 2 and Table 3 and described in Section 3. (ii) Even if there is no ortholog, the same principles apply: for example, even if there is no MTF1 ortholog, there is still the need for adaptation to Cu excess; even if there is no CCS ortholog, there is still a mechanism required for loading Cu-dependent SODs. (iii) Underneath all these reasons specific to Cu homeostasis, there are, of course, general reasons for using *C. elegans* as an animal model of biological processes in humans, such as, among others, its simple structure as a multicellular organism, its short lifespan, its transparency and the simplicity of genetic manipulation.
